# Perspectives on the Origin of Biological Homochirality on Earth

**DOI:** 10.1007/s00239-019-09897-1

**Published:** 2019-06-28

**Authors:** Koji Tamura

**Affiliations:** 1 Department of Biological Science and Technology, Tokyo University of Science, 6-3-1 Niijuku, Katsushika-ku, Tokyo, 125-8585 Japan; 2 Research Institute for Science and Technology, Tokyo University of Science, 2641 Yamazaki, Noda, Chiba 278-8510 Japan

**Keywords:** Amino acid, tRNA aminoacylation, Evolution, Homochirality, Origin of life, RNA world

## Abstract

The origin of biological homochirality on Earth has been an important unresolved issue in the field of molecular evolution and many hypotheses have been proposed to explain this. The most prevailing view may be that of astrobiologists, in that a slight enantiomeric excess of l-amino acids in meteorites can account for the origin. However, the view ignores two important factors: amino acid racemization, and the evolution and continuity of biological systems on Earth. Therefore, on the basis of these two standpoints, the plausibility of the hypothesis that chiral-selective tRNA aminoacylation could have led to crucial homochiral protein biosynthesis should be emphasized. Recent molecular dynamic simulations have clearly elucidated the mechanisms of enantiomer-specific aminoacylation. These studies strengthen the possibility that the hypothesized chiral selection of amino acids in biological systems actually occurred at the molecular level. It is significant to raise the points because the topic so far has tended to be expressed unclearly and ambiguously and also handled as such owing to its very nature.

The successful touch down of Japan’s Hayabusa2 space probe on the Ryugu asteroid located 340 million kilometers from Earth is expected to lead to the discovery of molecular components of life, e.g., amino acids (Castelvecchi 2019). However, as Stuart Kauffman pointed out using a metaphor with buttons and threads, the emergence of life is extremely hard to attain until a critical threshold toward phase transition is reached in the complex system (Kauffman 1996). Furthermore, life on Earth is intrinsically characterized by the existence of chiral features, and the origin of homochirality of natural amino acids and sugars remains an especially intriguing mystery (Atencio 2012). Natural proteins comprise α-amino acids that are exclusively left-handed (l-amino acids), whereas DNA and RNA contain right-handed sugars (d-deoxyribose and d-ribose, respectively). Here, the author would like to propose a perspective on the origin of homochirality, based on the molecular evolution of life on Earth.

The structural requirements of proteins and nucleic acids depend on their homochirality of their components: the α-helices and β-sheets of the secondary structures of proteins are likely to be formed only if the constituent amino acids are homochiral (all-l or all-d) (Bonner 2000). These secondary structures become the basis for constructing the proper tertiary structures of proteins, which confer specific roles on each protein. Similarly, template-directed elongation of nucleotide chains occurs properly only when the ribose sugars of the template are homochiral, and when the ribose sugar of each added mononucleotide has the same chirality as that of the template: poly-C-directed polymerization of activated guanosine (G) mononucleotide (5′-phosphorimidazolide) occurred only when C and G shared the same chirality (Joyce et al. 1984). Clay mineral (montmorillonite) catalyzes the oligomerization of 5′-phosphorimidazolides of nucleosides, yielding the corresponding oligonucleotides (Ferris and Ertem 1992, 1993; Kawamura and Ferris 1994). Montmorillonite-catalyzed oligomerization of racemic 5′-phosphorimidazolide of adenosine proceeds in a homochiral selective manner to preferentially yield homogeneous l- and d-oligomers (Joshi et al. 2000; Urata et al. 2001). Although these observations illustrate the importance of “homo” chirality, it is not clear why natural proteins and natural nucleic acids are composed of l-amino acids and d-sugars, respectively.

In elementary particle physics, parity violation during β-decay of nuclei may have led to a slight increase in the ratio of l- to d-enantiomers (< 10^−11^) (Hegstrom 1987). It is also suggested that polarized synchrotron radiation from neutron stars may have influenced the proportions of the 2 enantiomers (Bonner 2000). Furthermore, a spatially extended high circular polarization region was discovered around the massive star-forming region, the BN/KL nebula; subsequently, the possibility of meteorites delivering l-amino acids on the early Earth and leading to the initial bias in the ratio has been discussed (Fukue et al. 2010). In fact, l-amino acids have been found in only slightly greater proportions than d-amino acids in some real meteorites (Chyba et al. 1990). However, a slight enantiomeric excess of amino acids would be degraded by racemization. The half-lives of amino acid racemizations are expected to be 10^5^–10^6^ years at temperatures characteristic of the Earth’s surface (Bada and Miller 1987). The estimated half-life for free aspartic acid in aqueous solution (100 °C, pH 7–8) is just 30 days (Bada and Miller 1987). As a model of the silicon cycle in the Precambrian era shows, seawater temperature in the primitive Earth would have been much higher than today (Robert and Chaussidon 2006), although the temperature reconstruction has been debated in considering the various origins of cherts (Marin-Carbonne et al. 2014). Therefore, the racemization of amino acids should be primarily considered. In addition, l-isoform predominance was found in α-methyl amino acids in meteorites, but not in α-H amino acids (natural components of proteins) that are more easily racemized (Cronin and Pizzarello 1997). In terms of chemical reactions, autocatalysis can lead to a small initial enantiomeric excess of a chiral molecule, as seen in the case of 5-pyrimidyl alkanol treated with diisopropylzinc and pyrimidine-5-carboxaldehyde (Soai et al. 1995), but it is not directly related with the biological enantiomeric excess of l-amino acids.

Therefore, the utilization of homochiral molecules must have occurred at the time of the origin of life or shortly thereafter, with positive involvement of related molecules constituting reaction systems. The discovery of ribozymes solved the classic “chicken-or-egg” conundrum in molecular biology, and the “RNA world” could have existed during the initial stages of the formation of life (Gilbert 1986). Transition from the putative “RNA world” to the “protein world” was the key step in the establishment of life on Earth (Tamura 2015). In that sense, the interaction of RNA and amino acids could have determined the biological homochirality. The chirality of RNA has actually been shown to correlate with recognition of chiral amino acids through tRNA aminoacylation. Aminoacyl-tRNAs are key substrates for peptide bond formation in protein synthesis. A simple, minimized non-enzymatic aminoacylation model that captures the essence of the interactions seen in modern biological translation systems discovered this important feature: d-ribose RNA aminoacylates l-amino acids, whereas l-ribose RNA aminoacylates d-amino acids. The design was based on the rationale that an aminoacyl phosphate oligonucleotide hybridizes to the 3′-end of the RNA minihelix through a bridging oligonucleotide, thereby bringing together the activated amino acid and the amino acid attachment site (Fig. 1a) (Tamura and Schimmel 2004). Significantly, recent molecular dynamics (MD) simulations have clearly elucidated enantiomer-specific aminoacylation mechanisms after many years without success (Fig. 1b, c) (Ando et al. 2018). Atomic-scale geometric considerations for aminoacylation reactions include (1) Distance between an R-O^−^ nucleophile (Nu) of 3′-OH of the terminal adenosine of the RNA and carbonyl carbon of aminoacyl phosphate; (2) Bürgi–Dunitz (BD) angle; (3) Flippin–Lodge (FL) angle; (4) Lobe angle; (5) Distance between Nu and non-bridging phosphate oxygens (Fig. 1b) (Ando et al. 2018). The MD trajectories showed that the structures surrounding the active site of the system were formed close to the ideal A-form RNA double helix, where the distance between the 3′-O (nucleophile) and the carbonyl carbon (nucleophilic center) was shorter than that between the 2′-O and the carbon. Thus, the probability of forming a reactive geometry with 3′-O was much higher than that with 2′-O (Ando et al. 2018). Similar to the inter-transfer of amino acids, a stereoselective intramolecular aminoacylation process has also been demonstrated previously (Wickramasinghe et al. 1991; Liu et al. 2019). These studies strengthened the plausibility of the hypothesis that chiral-selective tRNA aminoacylation could have led to crucial homochiral protein biosynthesis.

**Fig. 1:**
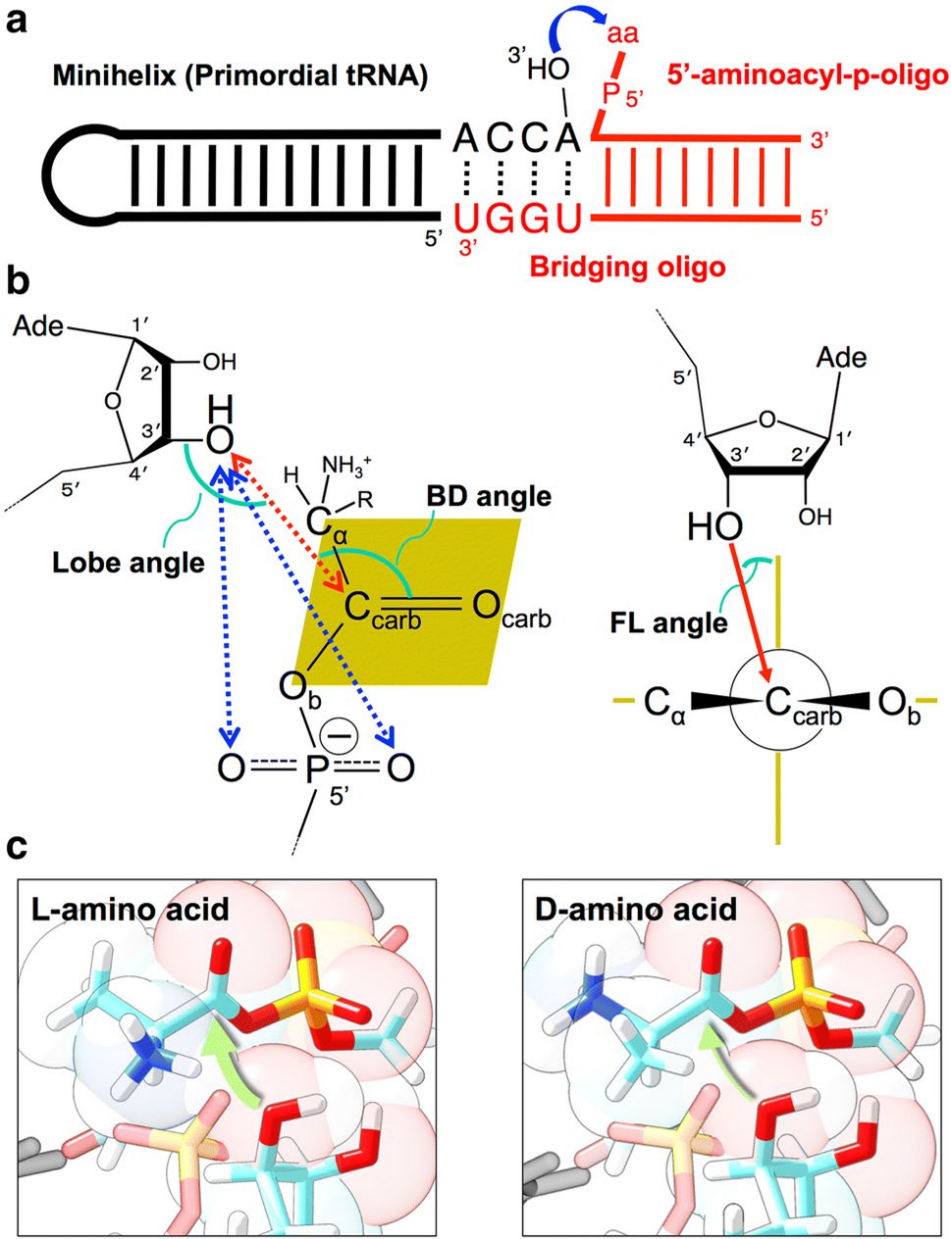
**a** Scheme for chiral-selective aminoacylation of an RNA minihelix (primordial tRNA) with an aminoacyl phosphate oligonucleotide and a bridging oligonucleotide (Tamura and Schimmel 2004). **b** Details of the aminoacylation reaction. Angles and distances defining the geometry are shown (Ando et al. 2018). These are (1) Distance between an R-O^−^ nucleophile (Nu) of 3′-OH of the terminal adenosine of the RNA and carbonyl carbon of aminoacyl phosphate; (2) Bürgi–Dunitz (BD) angle; (3) Flippin–Lodge (FL) angle; (4) Lobe angle; (5) Distance between Nu and non-bridging phosphate oxygens. **c** Structures of the reaction site in l-amino acid (alanine) (left) and d-amino acid (alanine) (right) obtained by molecular dynamics simulation (Ando et al. 2018). The green arrows indicate the nucleophilic attack of the oxygen of the 3′-OH on the carbonyl carbon of aminoacyl phosphate. In the case of d-amino acid, the side chain of the amino acid was placed close to the 3′-OH, making it difficult for nucleophilic attack to occur sterically

Such interaction specificity between RNA and amino acids may alternatively lead us to the hypothesis that l-amino acids selected d-ribose RNAs. However, ribose has 4 asymmetric centers (C_1′_, C_2′_, C_3′_, and C_4′_), and Watson–Crick helices can be formed properly if any 2 of the 3 asymmetric carbons (C_1′_, C_3′_, and C_4′_) are correctly positioned relative to each other (Bada and Miller 1987). If we assume the existence of RNA in its present day form (chain of d-riboses with proper configurations linked through 5′–3′ phosphodiester bond) a priori, chiral selection for l-amino acids by RNA would be strongly supported, while the probability that l-amino acids could have selected d-ribose RNAs with proper configurations would be quite low, considering the complex set of asymmetric centers in ribose. These scenarios would strongly support the RNA-directed selection of homochiral amino acids, and disfavor the alternative hypothesis that amino acid homochirality determined RNA homochirality (Tamura 2009). Once the d-ribose-based RNA world was established, l-amino acids could have been selected through the process of tRNA aminoacylation.

Template-directed auto-oligomerization was performed using all possible combinations of homochiral and heterochiral diastereomers of pyranosyl-RNA tetramer with 2′,3′-cyclophosphates (Bolli et al. 1997). As the chains elongated, the oligomerization proceeded by chiral selectively and produced homochiral products (Bolli et al. 1997). In principle, the chiral specific l- and d-libraries consist of equal amounts of homochiral all-l and all-d oligomers. However, the number of possible sequences is beyond the number of sequences actually formed, i.e., any given sequence in both libraries would actually occur only once. Thus, the sequence composition of these two libraries is not identical. If one specific RNA in the d-library exhibits a chemical property that is favorable for the evolution of the biological system, the d-ribose-based RNA world would be selected (Tamura 2008).

How did the original selection for d-ribose occur? Was it merely a coincidence or was it driven by necessity? Chiral amino acids may have served as potential asymmetric catalysts for the formation of sugars. The influences of non-racemic alanine and isovaline, which are also contained in meteorites, on the formation of sugars (tetroses) have been examined, and their chiral configurations have been found to be affected by the chirality of the amino acid catalysts (Pizzarello and Weber 2004). Such a process may have induced a chiral selection of d-ribose as a component of the RNA backbone. Furthermore, circular dichroism spectra of achiral diprotonated porphyrins in aqueous solution showed that the supramolecular association of the porphyrins is dependent on the rotation of the vortex direction (Ribó et al. 2001). The observation is interpreted in terms of hydrodynamic and steric effects, and the folding of the neighboring modified porphyrin chains in the same direction is thought to result in better stacking of the homoassociates (Rubires et al. 2001). It is also speculated that vortex-induced similar interactions may be caused in the case of nucleotides in unknown experimental conditions. If this theory is proved, the role of different hemispherical vorticities related to the origin of biological chirality can be speculated (Mason 1984).

Thus, possible selective forces may include Earth’s rotation around its own axis, light from the sun, geothermal energy, and pressure in thermal vents. A slight enantiomeric excess of amino acid could not account for the origin of biological homochirality. Rather, this riddle of biological homochirality may be solved only when we consider causative factors within the context of the entire Earth.
