# Correction: Perspectives on the Origin of Biological Homochirality on Earth

**DOI:** 10.1007/s00239-024-10206-8

**Published:** 2024-09-26

**Authors:** Koji Tamura

**Affiliations:** 1 Department of Biological Science and Technology, Tokyo University of Science, 6-3-1 Niijuku, Katsushika-Ku, Tokyo, 125-8585 Japan; 2 Research Institute for Science and Technology, Tokyo University of Science, 2641 Yamazaki, Noda, Chiba 278-8510 Japan

**Journal of Molecular Evolution (2019) 87:143–146** 10.1007/s00239-019-09897-1

The article Perspectives on the Origin of Biological Homochirality on Earth, written by Koji Tamura, was originally published electronically on the publisher’s Internet portal on 28 June 2019 without open access. With the author(s)’ decision to opt for Open Choice the copyright of the article changed on 29 August 2024 to © The Author(s) 2024 and the article is forthwith distributed under a Creative Commons Attribution 4.0 International License, which permits use, sharing, adaptation, distribution and reproduction in any medium or format, as long as you give appropriate credit to the original author(s) and the source, provide a link to the Creative Commons licence, and indicate if changes were made. The images or other third party material in this article are included in the article’s Creative Commons licence, unless indicated otherwise in a credit line to the material. If material is not included in the article’s Creative Commons licence and your intended use is not permitted by statutory regulation or exceeds the permitted use, you will need to obtain permission directly from the copyright holder. To view a copy of this licence, visit http://creativecommons.org/licenses/by/4.0.

